# 

**Published:** 1917-10-06

**Authors:** 


### Manager's Notices.

**Now Ready, "The Hospital" Index to Vol. LXI., Oct. 1916 to April 1917. Price 2½d. per copy post free**

**All letters on business matters must be addressed to The Manager, "The Hospital," 28 and 29 Southampton Street, London, W.C. 2.**

**Subscriptions**, which may commence at any time, are payable in advance. Cheques and Post-Office Orders should be crossed London County and Westminster Bank, Covent Garden Branch, and made payable to the Manager of The Hospital.

Rates of Subscription (payable in advance).

| | | |
|---|---|---|
| **UNITED KINGDOM.** | | |
| Three Months | (including Postage) | 2s. 0d |
| Six Months | do. | 4s. 0d |
| Twelve Months | do. | 6s. 6d. |
| **FOREIGN AND COLONIAL.** | | |
| Three Months | (including Postage) | 2s. 6d. |
| Six Months | do | 5s. 0d. |
| Twelve Months | do. | 8s. 8d. |

### Alexandra Day.

On September 26, the Administrative Committee of the "Alexandra Day" Fund met at the Mansion House to distribute the sum of £22,000 collected in London this year. Grants indicated by Queen Alexandra, who sent her thanks to the organisers, were announced amounting to £8,080. The details of the allocation of the remaining portion of the fund have not yet been made public.

### The League of Mercy.

A League of Mercy War Bazaar is to be held at the Ritz Hotel, Piccadilly, on October 24 and 25 next, on behalf of the hospitals. The event is being organised by the Presidents and Lady Presidents of certain districts of the League of Mercy. The official announcement states that the proposal has received the approval of the Grand President, Brigadier-General the Earl of Athlone, G.C.B., G.C.V.O., D.S.O., and the Lady President H.R.H. Princess Alice (Countess of Athlone), under the exceptional circumstances due to the war. The bazaar will be opened on the first day by a member of the Royal Family. All information can be had at the League of Mercy offices, 29 Southampton Street, Strand, or from the Hon. Organising Secretaries, Mr. W. and Miss Andrews, 5 Belsize Park, Hampstead.

### Matrons' and Sisters' Appointments.

Royal Infirmary, Gloucester.—Miss E. M. Garrick has been appointed assistant matron. She was trained at the London Hospital, and has since been sister at the Kasr-el-Aini Hospital, Cairo, at the English Hospital, Rio de Janeiro, and at the Queen's Hospital for Children, Hackney Road. She holds the certificate of the Central Midwives Board.

Bagthorpe Infirmary, Nottingham.—Miss Ada Stacey has been appointed ward sister. She was trained at North Evington Poor-Law Infirmary, Leicester, where she has since been ward sister.

Bedford County Hospital.—Miss Nancy Prevost has been appointed theatre sister. She was trained at the Royal Sussex County Hospital, Brighton.

Dundee War Hospital.—Miss Winifred Huebner has been appointed sister. She was trained at Camberwell Infirmary, and has since been at Wayland Military Hospital, Norfolk, and night sister at the Battersea General Hospital.

Fishmongers' Hall Hospital for Officers, London Bridge.—Miss Ella Young has been appointed sister. She was trained at Hull Royal Infirmary, and has been sister at York County Hospital. She has also seen war service in Belgium.

Royal Sussex County Hospital, Brighton.—Miss G. A. Smith has been appointed night sister. She was trained at St. Bartholomew's Hospital, E.C., and for the last two and a half years has been a member of Queen Alexandra's Imperial Military Nursing Service Reserve.

**NOTE.—Particulars of Appointments for insertion in this column will be welcomed.**

